# Queen's Hospital, Birmingham

**Published:** 1852-04

**Authors:** W. J. Moore

**Affiliations:** House-Surgeon.


### ARTICLE VIII.

*Queen's Hospital, Birmingham. Reported by* W. J. Moore, Esq., House-Surgeon.

*Dislocation and Fracture of the Lower Jaw.*—When the lower jaw is fractured, it is often a matter of some difficulty to keep the broken parts in apposition, on account of the strength of the muscles attached, and the strain caused on the parts by their spasmodic action. When the accident occurs to a child, the difficulty is ten-fold increased; and it is more than probable in such a case, that union without deformity will not occur; also, when there is dislocation as well as fracture it renders the case an awkward complication. Of course, the more approved method is that of fastening the teeth together, which, with the help of suitable bandages, generally succeeds; but it occasionally happens that this plan will not do, and that the posterior portion is pulled towards the centre of the mouth in spite of all; then, in such a case, a piece of wood passing transversely across the mouth and propping the teeth has proved of great service.

Among other cases, the following have been treated at this hospital.

Edward Cregoe, aged 14, was admitted, under the care of Mr. Sands Cox, on the 15th of October, having received a kick from a horse on the side of the head and face. There were present, pale features, feeble pulse, and other symptoms of partial collapse. He was, however, sensible, and could readily answer questions. The mouth was found open, the chin slightly protruding and somewhat turned to the right side; there was also much swelling about the zygoma and articulation of that side. As it was evident a dislocation existed, pressure with the thumbs was had recourse to; and, after some manipulation, the jaw appeared returned to its proper position. There was, however, no noise heard during the reduction, but he evidently could move it in a greater degree. After the application of suitable bandages, he was put to bed where he remained some hours before he quite recovered from the collapse consequent on the accident.

The next day the swelling of the side of the face was much increased; but, on examination, Mr. Sands Cox detected crepitus near the upper portion of the perpendicular ramus. This fact, however, required no modification in the treatment, and the boy went on favorably, the only medicines requisite being a dose or two of calomel during the stage of reaction.

He ultimately left the hospital on October 24, feeling his jaw nearly well; and in the course of another week, again presented himself, able to bite the hardest crust with ease.

The features of interest in this case are—1st. That the fracture occurred in the perpendicular portion of the bone, the horizontal ramus being by far the most frequent seat. 2nd. The occurrence of dislocation of one condyle only, dislocation of both being generally present; and 3rd. The occurrence of dislocation in conjunction with fracture. Here, however, no modification of treatment was necessary; but, had the fracture occurred in some other portion of the bone, the treatment would probably have been attended with more difficulty.

*Compound Fracture of the Lower Jaw.*—Patrick Welsh, aged 40, admitted June 24th, under the care of Mr. Knowles. He had half an hour before received a kick on the lower jaw, which caused a fracture, oblique in direction of the horizontal ramus of the left side. The mucous membrane was lacerated much, and one of the teeth lost, while the broken parts were more than half an inch separate. There was also a good deal of bleeding. This having been stopped, the parts were brought into apposition, and with much difficulty kept in place by means of thin strong wire passing round the adjacent teeth, aided by a piece of wood passing across the mouth, from the first molar on the right side to the first tooth of the fractured portion on the left, thus propping the parts in position. A gutta percha support was also adjusted beneath the jaw, and a four-tailed bandage used. He went on well, requiring only a dose of opening medicine. There was swelling beneath the tongue for a few days, but that decreasing, he was eventually made an out-patient, on July 12th, stating that he could eat meat and crust with facility. The support, however, was not taken away for some days afterwards.

In treating such cases, some dexterity is required in the adjustment of the wire, lest, by pressure, ulceration of the gums take place. It is well to use two or three rounds of wire, passing them, not only between the two teeth next to the fractured portion, but also between the second and third from that part, in order that the stress should not all rest on one tooth, and that tooth often shaken in its socket by the violence which produces the fracture. It is also worthy of notice in how short a space of time, fractures of the jaw will become firm, which evidently depends on the vascularity of the part.

*Compound Fracture of Lower Jaw in a Child.*—Bridget Varney, admitted August 3, under the care of Mr. Knowles. A short time previously she had been run over, and there was a large scalp wound extending over the right parietal bone, and a fracture oblique in direction of the right horizontal ramus of the lower jaw. There was also extensive laceration of the soft parts, and the fractured portions were some distance apart. One tooth, the last molar, was deficient, and the fracture extended from its site obliquely backwards towards the angle of the jaw. Under these circumstances, no retentive means could be employed within the cavity of the mouth; and, indeed, had this been possible, the crying and struggling of the child would have rendered it a matter of no little difficulty. External means were therefore had recourse to, and a very strong piece of gutta percha applied, which, with a bandage, kept the parts somewhat in apposition. Still, however, they could not be accurately adjusted; that is to say, they would not remain so. The child, however, speedily recovered, and now has but little deformity, and that little evidently disappearing as she grows older. The scalp wound was some time healing after the jaw was quite strong.—*Med. Times.*

